# Roles of Nuclear Orphan Receptors TR2 and TR4 during Hematopoiesis

**DOI:** 10.3390/genes15050563

**Published:** 2024-04-27

**Authors:** Greggory Myers, Yanan Sun, Yu Wang, Hajar Benmhammed, Shuaiying Cui

**Affiliations:** 1 Departments of Cell and Developmental Biology, University of Michigan Medical School, Ann Arbor, MI 48105, USA; mrgregg@umich.edu (G.M.); kxlnavpq@umich.edu (Y.W.); 2 Section of Hematology-Medical Oncology, Department of Medicine, Boston University Chobanian & Avedisian School of Medicine, Boston Medical Center, Boston, MA 02118, USA; ysun3@bu.edu (Y.S.); hajarben@bu.edu (H.B.)

**Keywords:** nuclear receptor, TR2, TR4, hematopoiesis, fetal hemoglobin, erythropoiesis

## Abstract

TR2 and TR4 (NR2C1 and NR2C2, respectively) are evolutionarily conserved nuclear orphan receptors capable of binding direct repeat sequences in a stage-specific manner. Like other nuclear receptors, TR2 and TR4 possess important roles in transcriptional activation or repression with developmental stage and tissue specificity. TR2 and TR4 bind DNA and possess the ability to complex with available cofactors mediating developmental stage-specific actions in primitive and definitive erythrocytes. In erythropoiesis, TR2 and TR4 are required for erythroid development, maturation, and key erythroid transcription factor regulation. TR2 and TR4 recruit and interact with transcriptional corepressors or coactivators to elicit developmental stage-specific gene regulation during hematopoiesis.

## 1. Introduction

Nuclear receptors (NRs) are a family of ligand-regulated transcription factors that act as precisely controlled activators or repressors based on their ability to bind small target ligands. NRs feature a highly conserved DNA-binding domain (DBD), a variable N-terminal regulatory domain (NTD) that contains an activation function (AF-1), a hinge region, and a ligand-binding domain on the C-terminus which may mediate dimerization or interaction partner binding. The ligand-binding domain (LBD) retains an activation function (AF-2) mediating the action of ligand binding [1].

NR families are divided based on the phylogeny of the most prominent members, as follows [2]:

I. Thyroid hormone receptor-like, which includes non-steroid hormone receptors thyroid hormone (TR), retinoic acid receptors (RARs), peroxisome proliferator-activated receptor (PPAR), and vitamin D receptor-like (VDR).

II. Retinoid X receptor-like, which includes retinoid x receptor (RXR), chicken ovalbumin upstream promoter transcription factors (COUP-TFs), and Testicular receptors (TR2 and TR4).

III. Estrogen receptor-like, which includes estrogen and androgen receptors (ER and AR), as well as glucocorticoid and mineralocorticoid receptors (GR and MR).

Testicular receptors 2 and 4 (TR2/TR4) were discovered in 1988 and 1994, respectively, and are expressed at similar levels in most major tissues, such as the kidney, brain, intestine, and liver, as well as in spermatocytes and erythrocytes [3,4,5,6,7,8,9]. TR2 and TR4 have roles in central nervous system development, lipid regulation, spermatogenesis, erythropoiesis, diabetes, and cancer initiation and progression [5,8,9,10,11,12,13,14,15]. This review attempts to summarize the most recent advances in TR2 and TR4 functions during hematopoiesis without discussing their roles in cancer and other diseases.

## 2. TR2/TR4 Structure and Putative Ligands

**Figure 1:**
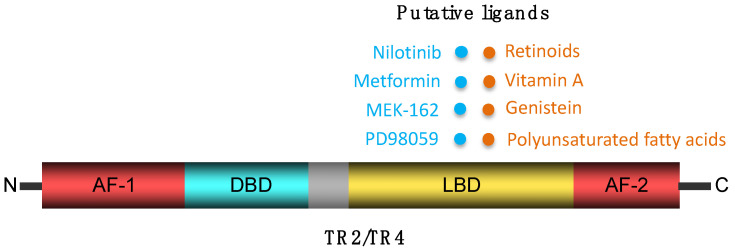
TR2 and TR4 function domains and putative ligands. AF, activation function; DBD, DNA-binding domain; LBD, ligand-binding domain.

Like other NRs, both TR2 and TR4 have several function domains. The N-terminal DBD can recognize and bind to specific DNA sequences. The LBD near the C-terminal enables the binding of potential ligands, such as hormones or small molecules, which may induce conformational changes in TR2 and TR4 and affect their transcriptional activity. AF domains might be responsible for target gene activation or repression by recruiting either coactivator or corepressor proteins to the transcription initiation complex (Figure 1).

Most NRs homo- or heterodimerize with another NR before binding two copies of a directly repeated (DR) hexanucleotide sequence called a half-site. The half-site consensus sequence for TR2 and TR4 is AGGTCA and occurs in variable orientations, such as the sequencing being everted or inverted [3,4]. The two half-sites may be separated by a variable number of nucleotide spacings, with a number indicating the nucleotide difference between them, for example, DR1 indicates one nucleotide between both directly repeated half-sites [16]. The variation of half-site orientation and spacing likely varies the affinity, which correlates with TR4 binding. Consistent with that notion, ChIP-seq and CUT&RUN analysis have revealed DR1 as the highest-represented DR element in erythroid cells [16].

TR2 and TR4 are classified as orphan receptors, meaning that their associated endogenous ligands have not been identified definitively. A few studies indicate that their activity can be influenced by a range of exogenous ligands and cofactors. While TR4 has been shown to respond to vitamin A, the concentrations at which it responds in vitro suggests this may not be the bona fide activating ligand [10]. A physical interaction-based surface plasmon resonance imaging assay has revealed that nilotinib demonstrated the most potent inhibition, whereas genistein emerged as the most potent activator of TR4 [17]. Other potential ligands, including polyunsaturated fatty acids, have also been shown to promote modest TR4 transcriptional activity when compared to the concentration necessary to induce activation of a variety of other known NRs [18,19]. Metformin and extracellular signal-regulated kinase (ERK) inhibitors, MEK-162 and PD98059, have been reported to inhibit TR4 transcriptional activity [20,21,22].

## 3. Hematopoiesis and Globin Switching

**Figure 2:**
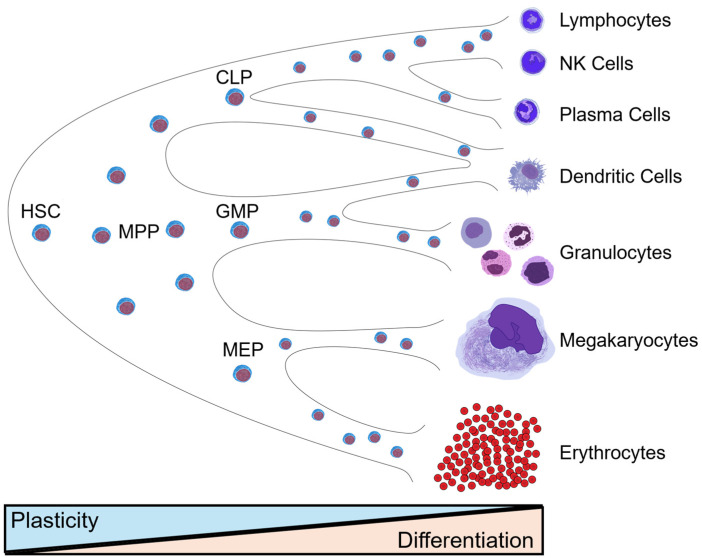
Model of hematopoiesis. HSCs retain high plasticity and produce MPPs which are totipotent with limited self-renewal capacity. The progeny of MPPs are increasingly lineage-restricted and responsible for the production of all differentiated blood cell types. HSC, Hematopoietic Stem Cell; MPP, Multipotent Progenitor; MEP, Megakaryocyte-Erythroid Progenitor; GMP, Granulocyte-Macrophage Progenitor; CLP, Common Lymphocyte Progenitor.

Within hematopoiesis, the roles of TR2 and TR4 have been highly characterized in erythropoiesis. In mammals, the first erythroid cells emerge from “blood islands” comprised of hemangioblasts within the yolk sac, which gives rise to the first wave of primitive erythroid progenitors. In mice, this wave of transient progenitors arises from the yolk sac around embryonic day 7.5 (e7.5), and wanes from e12.5–e16.5 to give way for definitive erythropoiesis occurring in the developing fetal liver [23]. The fetal liver is seeded by erythromyeloid progenitors (EMPs) derived from the yolk sac at e8.5, which produces the first wave of definitive erythrocytes to sustain embryogenesis until birth [24]. After birth, the primary site of erythropoiesis shifts to the bone marrow where hematopoietic stem cells (HSCs) take over as the primary source of all hematopoietic cells, including erythrocytes. HSCs initially specify a heterogeneous population of multipotent progenitors (MPPs), which have the potential to produce increasingly restricted pools of downstream progenitor populations, such as the megakaryocyte-erythroid progenitor (MEP), granulocyte-macrophage progenitor (GMP), or common lymphoid progenitor (CLP) (Figure 2). The final stage in commitment to the erythroid lineage occurs when MEPs differentiate into a functionally defined burst-forming unit (BFU-E) [25]. BFU-E possesses the bulk of the proliferative potential of erythroid cells and ultimately differentiates to produce smaller functionally defined erythroid precursors known as colony-forming units (CFU-E) [26]. Each CFU-E differentiates to produce a proerythroblast, which undergoes successive symmetric divisions to generate basophilic, polychromatic, and orthochromatic erythroblasts, doubling the number of cells at each stage. Orthochromatic erythroblasts undergo enucleation to produce reticulocytes that mature to erythrocytes as they enter circulation.

While circulating erythrocytes may have different developmental origins during embryogenesis, depending on which wave of erythropoiesis is responsible for their production, so too does the content of their hemoglobin differ depending on their developmental origins. Hemoglobin is comprised of two subunits, namely an α-like subunit and a β-like globin gene subunit. The human α- and β-globin loci each hold four and five different globin genes, respectively, while the murine α- and β-globin loci have three and four different respective globin genes. These genes are expressed in order during development under the control of the 5′ locus control region (LCR) for each globin gene cluster. The LCR acts as a super-enhancer in conjunction with transcription factors that are recruited to the promoter of each globin gene to activate or repress each respective subunit, thereby controlling the timing and levels of globin gene expression [27,28,29].

**Figure 3:**
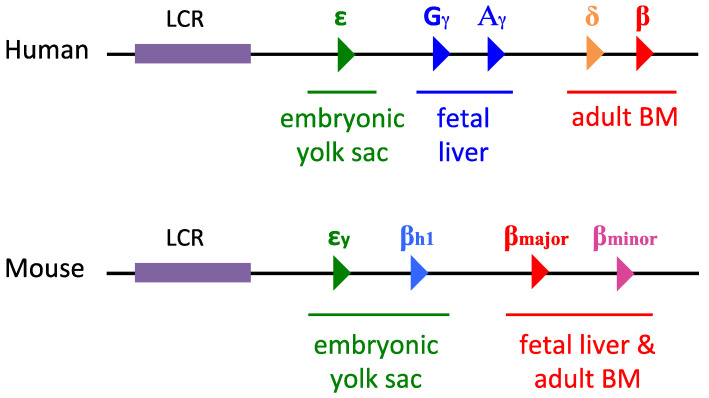
Structure and organization of human and mouse β-type globin loci. The LCR super-enhancer drives the developmentally coordinated β-type globin genes in order of their arrangement in the loci, beginning with the embryonic-type globin genes (ε in humans or εy/βh1 in mice) and progressing through the fetal globin genes (G_γ_ and A_γ_) to express the definitive β-globin genes (δ/β in humans or βmajor/βminor in mice) after birth. LCR, Locus control region; BM, Bone marrow.

At the human β-globin locus, the first β-like globin gene that is expressed is the embryonic ε-globin gene, and it is expressed in primitive erythroid progenitors derived from the yolk sac. To meet the oxygen demands of the developing fetus, the first switch in β-globin transcription results in the silencing of ε-globin and concomitant activation of the fetal γ-globin genes as the production of erythrocytes shifts to the fetal liver, marking the beginning of definitive erythropoiesis. Fetal hemoglobin (HbF) consisting of two α-globin chains and two γ-globin chains (α2γ2) has a greater affinity for oxygen than adult hemoglobin, thus enabling sufficient transfer of oxygen across the placenta. Gradually, at around the time of birth, a second switch from fetal γ-globin to adult β-globin transcription occurs as the site of hematopoiesis shifts again to the adult bone marrow (Figure 3). Shortly after birth, adult β-globin becomes the predominant form of globin subunit used in hemoglobin synthesis. The molecular mechanisms underlying the lineage and stage-specific globin switches through chromatin modifications have made the β-globin locus an attractive model for the dynamic role of epigenetics in gene regulation [30].

Mice are a commonly studied model due to their similarity in β-globin switching compared to humans. Mice express εy- and βh1-globin genes in primitive erythrocytes, until the βh1-globin gene is silenced, while the εy-globin gene persists as the adult β-globin genes (β_major_ and β_minor_) become the predominantly expressed and incorporated β-globin subunit near parturition (Figure 3).

## 4. Role of TR2/TR4 in Erythropoiesis

TR2 and TR4 are constitutively expressed throughout definitive erythroid differentiation [31,32]. The genetic requirements of TR2 and TR4 have been revealed through extensive use of loss-of-function mouse models. TR2 null mice display no obvious phenotype, while TR4 mice display growth and behavioral defects [9,33,34,35]. Compound mutant embryos lacking both TR2 and TR4 die preimplantation, initially suggesting the roles of TR2/4 may be partially redundant [36,37]. However, the complete loss of TR4 in congenic C57BL/6 background led to embryonic lethality before e9.5 of gestation, while partial loss led to defects in erythropoiesis [38].

The role of TR2 and TR4 in erythrocytes was first discovered while searching for the mechanisms that facilitate β-globin switching. Analysis of the β-globin locus has revealed DR sites in the promoter of ε- and γ-globin genes that, when mutated, lead to aberrant autonomous expression of either gene causing high-persistence of fetal hemoglobin (HPFH) [39,40]. Research investigating the phenomenon led to the isolation of TR2 and TR4 from erythroid cell lines in the form of proteins bound to the repressive DRED complex (Direct Repeat Erythroid-Definitive). Subsequently, TR2 and TR4 were shown to bind to DR1 sites in the ε- and γ-globin gene promoters in vitro using biochemical assays [33].

Experiments examining the role of TR2 and TR4 on globin expression in vivo by using human β-globin yeast artificial chromosome (β-YAC) transgenic mice (containing the entire human β-globin gene cluster) in conjunction with dominant-negative TR4 mice, demonstrated activation of human ε-globin in primitive and definitive erythrocytes as well as the activation of γ-globin in definitive erythroid cells, suggesting stage-specificity to the repressive roles of TR2 and TR4. Compound TR2 and TR4 mutants demonstrated induction of murine embryonic εy- and βh1-globin gene expression and the repression of the adult β_major_ gene [37].

Since compound TR2/TR4 null mice die peri-implantation, the role of TR2 and TR4 in erythropoiesis in vivo has been determined through conditional knockout experiments. In *Tr2*^−/−^ *Tr4*^f/f^ mice, isolated bone marrow progenitors were infected with a Cre-expressing adenovirus to delete TR4. After differentiation in an in vitro culture system that closely recapitulates erythroid development in vivo, loss of TR2 and TR4 attenuated differentiation and terminal maturation. While the cause of blocked differentiation is unclear, there was a significant loss in Kruppel-like Factor 1 (KLF1) and induction of GATA Binding Protein 1 (Gata1), both essential to erythroid development [37].

*Tr4*^−/−^ mice exhibited embryonic lethality prior to the onset of hematopoiesis [38]. Therefore, the role of Tr4 in murine erythropoiesis was studied in *Tr4*^+/−^ mice, which identified defects in erythroid differentiation and the proliferation of erythroid precursors. This is attributed to reduced expression of factors required for heme biosynthesis, such as Alas2 or Alad, and the increased expression of Cdkn1c, a factor which is responsible for negative regulation of cell-cycle progression [41].

The forced expression of TR2 and TR4 in sickle cell disease (SCD) mice has been shown to reduce pathological symptoms associated with the disease [42]. Subsequent studies attempting to ascertain the cause of the improvement have found evidence of a decrease in sickled erythrocytes adhering to vascular cell-adhesion molecule 1 (VCAM-1), which leads to vaso-occlusive events on endothelial cells [43]. The reduction in adherence may reflect either an increase in HbF or a reduction in circulating reticulocytes, which have greater properties of adherence than mature erythrocytes.

## 5. TR2/TR4 Interacting Corepressors

The DRED complex consists of a TR2/TR4 dimer as the DNA-binding scaffold, as well as cofactors DNA methyltransferase-1 (DNMT1) and lysine-specific histone demethylase 1 (LSD1) [44]. DNMT1 has been shown to recognize and methylate CpG dinucleotides opposite of MeCpG residues during DNA replication, allowing for epigenetic inheritance of DNA methylation essential for cell identity [45]. LSD1 can act as a transcriptional repressor by demethylating the activating marks mono- and dimethyl-histone H3 Lysine 4 (MeH3K4 and Me2H3K4) [46,47]. Alternatively, LSD1 may also act as a transcriptional activator in the context of other cofactors, such as androgen and estrogen receptors (AR and ER), by demethylating repressive marks mono- and dimethyl-histone H3 Lysine 9 (MeH3K9 and Me2H3K9) [48]. Recently, findings have shown that conditional loss of LSD1 or DNMT1 alters erythropoiesis, globin transcription, and corepressor binding on globin gene promoters [49]. Deficiency and chemical inhibition of LSD1 or DNMT1 reactivates γ-globin expression [49,50,51,52]. Preclinical studies of LSD1 inhibitors and DNMT1 inhibitors further support their roles in modulating HbF as a potential therapy to alleviate β-globinopathies [53,54].

TIF1β (also known as Trim28) was investigated subsequently following its copurification from TR2 and TR4 to determine what role it may play in β-globin gene expression and erythropoiesis due to its previous characterization of a transcriptional repressor in many cell types [55,56]. The TIF1β-HP1 system is responsible for maintaining HSC transcriptional integrity [57]. The in vivo loss of TIF1β in murine adult HSCs resulted in defective erythropoiesis, reduced heme biosynthesis enzymes, and increased apoptosis in immature erythrocytes [58].

Nuclear receptor corepressor-1 (NCoR1) was identified as the key component of the DRED complex through the use of proximity-dependent biotin identification (BioID), and serves as the direct adapter between TR2/TR4 and other corepressors [59]. The deubiquitinase BRCA1-associated protein-1 (BAP1) is responsible for maintaining NCoR1 at sites in the β-globin locus. Disruption of NCoR1 interaction with TR2/TR4 or deficiency of BAP1 reactivates γ-globin expression and induces HbF production.

Other interacting partners of TR2 and TR4 include histone deacetylase (HDAC) 1/2/3, CoREST, and the nucleosome-remodeling deacetylase (NuRD) complex (MTA1/2, Mi2B, RbAp, MBD2/3, and p66) [44,60]. TR2 and TR4 interact with CoREST and HDAC1/2 [44]. The association of LSD1, CoREST, and HDAC1/2 has been suggested to be responsible for histone deacetylation and H3K4 demethylation to bring about repressed chromatin states [46,61,62,63].

The NuRD complex has been shown to interact with LSD1 and DNMT1 to facilitate chromatin remodeling [64,65]. Numerous NuRD complex components, including MBD2, Mi2B (CHD4), and GATAD2A, have been shown to repress γ-globin expression directly or indirectly by disrupting the NuRD complex [55,66,67,68]. However, it is not clear whether interaction exists between these proteins and TR2 and TR4 as it pertains to γ-globin expression.

Those findings suggest that TR2 and TR4 may function as repressors in various capacities based on the properties of bound cofactors. Interestingly, the majority of the TR2/TR4 corepressors also interact with the BCL11A or ZBTB7A (LRF) [69,70,71,72], both of which are demonstrated to be the most physiologically significant γ-globin repressors, indicating that the corepressors may be recruited by multiple DNA-binding transcription factors to contribute to γ-globin repression. Whether different transcription factors interact with the same corepressors competitively remains to be elucidated.

## 6. TR2/TR4 Interacting Coactivators

A cell-based transfection assay system found that TR4 transcriptional activation is potentiated by PPARGC1A (PGC1α) and other members of the steroid receptor co-family (SRC1-3), with PGC1α being the most effective [10]. Subsequently, PGC1α and PGC1β were immunoprecipitated with TR2 and TR4. Of note, however, TR2 only bound PGC1α in erythrocytes, while TR4 bound both [73].

PGC1 compound mutant mice display hematological deficiencies such as anemia, thrombocytopenia, and leukopenia. The complete compound loss of PGC1 depletes globin gene expression at all stages and results in a block in terminal erythrocyte differentiation. During primitive erythropoiesis, the role of PGC1α and PGC1β appears to be compensatory since the loss of both genes has a more severe phenotype compared to the loss of either PGC1 variant. Conversely, in definitive erythroid cells there is no significant difference in globin gene expression in single PGC1 knockout versus compound mutant mice, indicating there is stage and gene specificity in its role of gene transcription.

In primitive erythroid cells, both PGC1α and PGC1β bind to εy- and βh1-globin promoters during times of abundant expression. In definitive erythroid cells, PGC1α/PGC1β associates the εy-globin gene promoter, while it is typically expressed and is not found at the repressed βh1 promoter or expressed β_major_ gene. At postnatal day 0, PGC1α and PGC1β do not bind with TR2 or TR4 at either promoter when the genes should be repressed. The concurrent binding of PGC1α and PGC1β in close proximity to TR2 and TR4 in strong correlation with the coordinated expression of εy- and βh1-globin genes strongly suggests their roles as stage-specific activators in association with TR2 and TR4 [73].

Similarly, in human primary erythroid progenitor CD34^+^ cells, an intriguing interaction between PGC-1α and TR4 was observed. Upregulation of PGC-1α in CD34^+^ cells using lentiviral overexpressing of PGC-1α or a PGC-1α agonist (ZLN005) leads to increased γ-globin expression at both mRNA and protein levels, as well as increased number of HbF positive cells (F-cells) during cultures [74]. These findings shed light on the potential regulatory roles of TR2/TR4 with the transcriptional co-activator PGC-1α in modulating γ-globin expression in human primary erythroid progenitor CD34^+^ cells.

## 7. TR2/TR4 Regulate Key Erythroid Genes

TR2 and TR4 play essential roles in the development of erythroid cells via their ability to regulate the expression of Gata1. Gata1 is an essential transcription factor required for the survival and differentiation of erythrocytes, megakaryocytes, and eosinophilic progenitors [75]. In erythropoiesis, Gata1 is responsible for the survival of erythroid precursors by activating erythropoietin signaling [76]. Loss of Gata1 results in embryonic lethality between e10.5–e11.5 due to anemia, as other studies have shown Gata1 null primitive and definitive erythroblasts undergo apoptosis [75,77,78,79]. TR2 and TR4 have been shown to directly bind the Gata1 hematopoietic enhancer (G1HE) −3.7 Kb upstream from the Gata1 1b exon and subsequently repress Gata1 during terminal erythroid differentiation [80]. It is hypothesized that TR2 and TR4 are responsible for the repression of Gata1 transcription in developing erythroblasts, a requirement thought to be essential for terminal erythroid maturation.

The compound loss of TR2 and TR4 in bone marrow progenitors leads to a loss of KLF1 expression, a transcription factor critical to erythroid development, and is itself activated by Gata1 [81,82]. KLF1 is required for the activation of the adult β-globin gene and may also function as a fetal globin gene repressor through activation of BCL11A or abrogation of repressors KLF3 and KLF8, null mutations of which cause increased fetal hemoglobin [82,83,84]. TR2/TR4 may also indirectly regulate KLF1 through interaction with Mi2B, a known activator of KLF1 and cofactor of TR2 and TR4 [55].

Reduction of TR4 abundance leads to decreased expression of Alad and Alas2 genes that are essential for heme biosynthesis and erythroid differentiation. In contrast, TR4 reduction results in increased expression of the proliferation inhibitory factor, cyclin-dependent kinase inhibitor (Cdkn1c). These findings support the key role of TR4 in promoting erythroid maturation and proliferation [38].

## 8. Summary and Future Perspectives

The dynamic landscape of chromatin throughout differentiation from the HSC requires precise and coordinated epigenetic changes which are mediated by nuclear receptors such as TR2 and TR4. TR2 and TR4 are capable of recruiting cofactors, and the interactions between them enable the chromatin remodeling required for differentiation. Loss of TR2 and TR4 results in the attenuation of erythrocyte differentiation, but further work is needed to elucidate the precise mechanism as TR2 and TR4 cannot be readily manipulated in vivo.

The regulatory targets of TR2 and TR4 are widely variable with regard to their necessity and function. Some targets, such as Gata1, are crucial to successful cell development, while others are required for cell metabolism. The multifaceted control of TR2 and TR4 target genes demonstrates the ability of nuclear receptors to allow for cell-specific differences in expression.

While TR2 and TR4 bind preferentially to DR1 sites, they possess the capability to bind half-sites with variable orientations and spacing. Alterations in NR-binding site affinity allow for multiple, simultaneous binding sites of TR2 and TR4 that may be occupied preferentially based on similarity to the consensus sequence. The abundance of NRs may determine the number and degree to which possible target genes are affected in a particular cell type.

**Figure 4:**
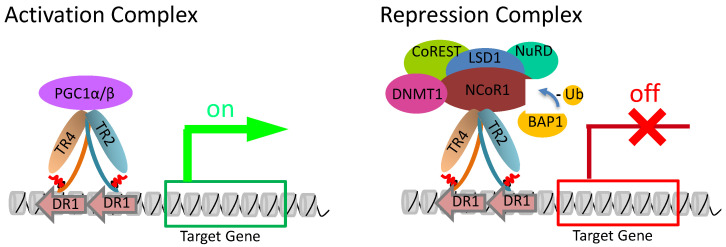
Model for TR2/TR4-mediated gene activation/repression. TR2 and TR4 bind to DR1 regions of DNA to form a scaffold for other coactivators (PGC1α/β) or corepressors (LSD1/DNMT1/NuRD/CoREST complex) to exert influences on gene expression. BAP1 deubiquitinates the adaptor protein NCoR1 to maintain the recruitment of corepressors.

To further increase the target diversity, interactions between transcription factor families allow for targets other than canonical binding sites. TR2 and TR4 interact with cofactor complexes to mediate target gene activation or repression. After nuclear receptors bind to their respective binding sites, the availability and the abundance of their cofactors determine whether the gene will be expressed. If coactivators are the dominant species of cofactor specific to an NR such as TR2 and TR4, then genes bound by them are activated. Conversely, should corepressors be the most available cofactor, then the NRs would mediate target gene suppression (Figure 4). The multitude of NR targets that require different patterns of expression can be mediated by one, if not more, regulatory mechanisms previously described.

The model in which the stage-specific molecular mechanisms cofactor recruitment may be studied at the β-globin locus to discern how cofactors may differentially act on genes throughout development from a stem-like progenitor. Targeted disruption of interactions between cofactors may allow for safer therapeutic interventions for blood diseases and hemoglobinopathies by leaving essential molecular interactions intact. More work is required to establish the precise interactions between TR2 and TR4 with known and as yet unknown cofactors to determine the mechanisms of control by this stage-specific gene regulator in developing erythroid cells. The findings from these studies might also help to establish therapeutic indications in other hematological malignancies, and help to understand the basic mechanisms of hematologic development related to cancer and other diseases.

## References

[1] Gronemeyer H., Gustafsson J.-A., Laudet V. (2004). Principles for modulation of the nuclear receptor superfamily. Nat. Rev. Drug Discov..

[2] Auwerx J., Baulieu E., Beato M., Becker-Andre M., Burbach P.H., Camerino G., Chambon P., Cooney A., Dejean A., Dreyer C. (1999). A Unified Nomenclature System for the Nuclear Receptor Superfamily. Cell.

[3] Chang C., Kokontis J. (1988). Identification of a new member of the steroid receptor super-family by cloning and sequence analysis. Biochem. Biophys. Res. Commun..

[4] Chang C., Da Silva S.L., Ideta R., Lee Y., Yeh S., Burbach J.P. (1994). Human and rat TR4 orphan receptors specify a subclass of the steroid receptor superfamily. Proc. Natl. Acad. Sci. USA.

[5] Lopes da Silva S., Van Horssen A.M., Chang C., Burbach J.P. (1995). Expression of nuclear hormone receptors in the rat supraoptic nucleus. Endocrinology.

[6] Lee H.J., Young W.J., Shih C.Y., Chang C. (1996). Suppression of the human erythropoietin gene expression by the TR2 orphan receptor, a member of the steroid receptor superfamily. J. Biol. Chem..

[7] Young W.J., Lee Y.F., Smith S.M., Chang C. (1998). A bidirectional regulation between the TR2/TR4 orphan receptors (TR2/TR4) and the ciliary neurotrophic factor (CNTF) signaling pathway. J. Biol. Chem..

[8] Liu N.-C., Lin W.-J., Kim E., Collins L.L., Lin H.-Y., Yu I.C., Sparks J.D., Chen L.-M., Lee Y.-F., Chang C. (2007). Loss of TR4 orphan nuclear receptor reduces phosphoenolpyruvate carboxykinase-mediated gluconeogenesis. Diabetes.

[9] Collins L.L., Lee Y.-F., Heinlein C.A., Liu N.-C., Chen Y.-T., Shyr C.-R., Meshul C.K., Uno H., Platt K.A., Chang C. (2004). Growth retardation and abnormal maternal behavior in mice lacking testicular orphan nuclear receptor 4. Proc. Natl. Acad. Sci. USA.

[10] Zhou X.E., Suino-Powell K.M., Xu Y., Chan C.-W., Tanabe O., Kruse S.W., Reynolds R., Engel J.D., Xu H.E. (2011). The orphan nuclear receptor TR4 is a vitamin A-activated nuclear receptor. J. Biol. Chem..

[11] Kang H.S., Okamoto K., Kim Y.S., Takeda Y., Bortner C.D., Dang H., Wada T., Xie W., Yang X.P., Liao G. (2011). Nuclear orphan receptor TAK1/TR4-deficient mice are protected against obesity-linked inflammation, hepatic steatosis, and insulin resistance. Diabetes.

[12] Parris T.Z. (2020). Pan-cancer analyses of human nuclear receptors reveal transcriptome diversity and prognostic value across cancer types. Sci. Rep..

[13] Lin S.J., Zhang Y., Liu N.C., Yang D.R., Li G., Chang C. (2014). Minireview: Pathophysiological roles of the TR4 nuclear receptor: Lessons learned from mice lacking TR4. Mol. Endocrinol..

[14] Zhang L.Y., Zhang J.Z., Ma Y.Y., Chen J.F., Dong B., Zhao W., Wang X., Zheng Q.F., Fang F., Yang Y. (2015). Testicular orphan receptor 4 (TR4) is a marker for metastasis and poor prognosis in non-small cell lung cancer that drives the EMT phenotype. Lung Cancer.

[15] Wang H., Luo W., Wang X., Xue D., Ren L., Xu L., Ge G., Xia L., Yu S., Wang M. (2021). Testicular Nuclear Receptor 4 Regulates Proliferation and Apoptosis of Bladder Cancer via Bcl-2. Front. Mol. Biosci..

[16] O’Geen H., Lin Y.H., Xu X., Echipare L., Komashko V.M., He D., Frietze S., Tanabe O., Shi L., Sartor M.A. (2010). Genome-wide binding of the orphan nuclear receptor TR4 suggests its general role in fundamental biological processes. BMC Genom..

[17] Xia L.Q., Shen D.Y., Wang H., Ren L.L., Chen Y., Li G.H. (2020). Identification of Small-Molecule Regulators of Testicular Receptor 4 via a Drug Repurposing Screening. ACS Omega.

[18] Xie S., Lee Y.-F., Kim E., Chen L.-M., Ni J., Fang L.-Y., Liu S., Lin S.-J., Abe J.-I., Berk B. (2009). TR4 nuclear receptor functions as a fatty acid sensor to modulate CD36 expression and foam cell formation. Proc. Natl. Acad. Sci. USA.

[19] Levin A.A., Sturzenbecker L.J., Kazmer S., Bosakowski T., Huselton C., Allenby G., Speck J., Kratzeisen C., Rosenberger M., Lovey A. (1992). 9-cis retinoic acid stereoisomer binds and activates the nuclear receptor RXR alpha. Nature.

[20] Kim E., Liu N.C., Yu I.C., Lin H.Y., Lee Y.F., Sparks J.D., Chen L.M., Chang C. (2011). Metformin inhibits nuclear receptor TR4-mediated hepatic stearoyl-CoA desaturase 1 gene expression with altered insulin sensitivity. Diabetes.

[21] Du L., Bergsneider M., Mirsadraei L., Young S.H., Jonker J.W., Downes M., Yong W.H., Evans R.M., Heaney A.P. (2013). Evidence for orphan nuclear receptor TR4 in the etiology of Cushing disease. Proc. Natl. Acad. Sci. USA.

[22] Zhang D., Bergsneider M., Wang M.B., Heaney A.P. (2016). Targeting the ERK pathway for the treatment of Cushing’s disease. Oncotarget.

[23] Palis J. (2014). Primitive and definitive erythropoiesis in mammals. Front. Physiol..

[24] Soares-da-Silva F., Freyer L., Elsaid R., Burlen-Defranoux O., Iturri L., Sismeiro O., Pinto-do O.P., Gomez-Perdiguero E., Cumano A. (2021). Yolk sac, but not hematopoietic stem cell-derived progenitors, sustain erythropoiesis throughout murine embryonic life. J. Exp. Med..

[25] Li J., Hale J., Bhagia P., Xue F., Chen L., Jaffray J., Yan H., Lane J., Gallagher P.G., Mohandas N. (2014). Isolation and transcriptome analyses of human erythroid progenitors: BFU-E and CFU-E. Blood.

[26] Li H., Natarajan A., Ezike J., Barrasa M.I., Le Y., Feder Z.A., Yang H., Ma C., Markoulaki S., Lodish H.F. (2019). Rate of Progression through a Continuum of Transit-Amplifying Progenitor Cell States Regulates Blood Cell Production. Dev. Cell.

[27] Choi O.R., Engel J.D. (1988). Developmental regulation of beta-globin gene switching. Cell.

[28] Tolhuis B., Palstra R.J., Splinter E., Grosveld F., de Laat W. (2002). Looping and interaction between hypersensitive sites in the active beta-globin locus. Mol. Cell.

[29] Carter D., Chakalova L., Osborne C.S., Dai Y.-F., Fraser P. (2002). Long-range chromatin regulatory interactions in vivo. Nat. Genet..

[30] Cui S., Engel J.D. (2017). Reactivation of Fetal Hemoglobin for Treating beta-Thalassemia and Sickle Cell Disease. Adv. Exp. Med. Biol..

[31] Shi L., Lin Y.-H., Sierant M.C., Zhu F., Cui S., Guan Y., Sartor M.A., Tanabe O., Lim K.-C., Engel J.D. (2014). Developmental transcriptome analysis of human erythropoiesis. Hum. Mol. Genet..

[32] Shi L., Sierant M.C., Gurdziel K., Zhu F., Cui S., Kolodziej K.E., Strouboulis J., Guan Y., Tanabe O., Lim K.-C. (2014). Biased, non-equivalent gene-proximal and -distal binding motifs of orphan nuclear receptor TR4 in primary human erythroid cells. PLoS Genet..

[33] Tanabe O., Katsuoka F., Campbell A.D., Song W., Yamamoto M., Tanimoto K., Engel J.D. (2002). An embryonic/fetal beta-type globin gene repressor contains a nuclear receptor TR2/TR4 heterodimer. EMBO J..

[34] Chen Y.-T., Collins L.L., Uno H., Chang C. (2005). Deficits in motor coordination with aberrant cerebellar development in mice lacking testicular orphan nuclear receptor 4. Mol. Cell. Biol..

[35] Mu X., Lee Y.-F., Liu N.-C., Chen Y.-T., Kim E., Shyr C.-R., Chang C. (2004). Targeted inactivation of testicular nuclear orphan receptor 4 delays and disrupts late meiotic prophase and subsequent meiotic divisions of spermatogenesis. Mol. Cell. Biol..

[36] Shyr C.R., Kang H.Y., Tsai M.Y., Liu N.C., Ku P.Y., Huang K.E., Chang C. (2009). Roles of testicular orphan nuclear receptors 2 and 4 in early embryonic development and embryonic stem cells. Endocrinology.

[37] Cui S., Tanabe O., Sierant M., Shi L., Campbell A., Lim K.C., Engel J.D. (2015). Compound loss of function of nuclear receptors Tr2 and Tr4 leads to induction of murine embryonic beta-type globin genes. Blood.

[38] Lee M.P., Tanabe O., Shi L., Jearawiriyapaisarn N., Lucas D., Engel J.D. (2017). The orphan nuclear receptor TR4 regulates erythroid cell proliferation and maturation. Blood.

[39] Fucharoen S., Shimizu K., Fukumaki Y. (1990). A novel C-T transition within the distal CCAAT motif of the G gamma-globin gene in the Japanese HPFH: Implication of factor binding in elevated fetal globin expression. Nucleic Acids Res..

[40] Gelinas R., Endlich B., Pfeiffer C., Yagi M., Stamatoyannopoulos G. (1985). G to A substitution in the distal CCAAT box of the A gamma-globin gene in Greek hereditary persistence of fetal haemoglobin. Nature.

[41] Pateras I.S., Apostolopoulou K., Niforou K., Kotsinas A., Gorgoulis V.G. (2009). p57KIP2: “Kip”ing the cell under control. Mol. Cancer Res..

[42] Campbell A.D., Cui S., Shi L., Urbonya R., Mathias A., Bradley K., Bonsu K.O., Douglas R.R., Halford B., Schmidt L. (2011). Forced TR2/TR4 expression in sickle cell disease mice confers enhanced fetal hemoglobin synthesis and alleviated disease phenotypes. Proc. Natl. Acad. Sci. USA.

[43] White J.C., Pawar A., Fu G., Cui S., Tavernier F., Hamid M., Harro D., Giacherio D., Campbell A.D., Hines P.C. (2015). TR2/TR4 overexpression in a humanized sickle cell disease mouse model decreases RBC adhesion to VCAM-1. Blood Cells Mol. Dis..

[44] Cui S., Kolodziej K.E., Obara N., Amaral-Psarris A., Demmers J., Shi L., Engel J.D., Grosveld F., Strouboulis J., Tanabe O. (2011). Nuclear receptors TR2 and TR4 recruit multiple epigenetic transcriptional corepressors that associate specifically with the embryonic β-type globin promoters in differentiated adult erythroid cells. Mol. Cell. Biol..

[45] Lei H., Oh S.P., Okano M., Jüttermann R., Goss K.A., Jaenisch R., Li E. (1996). De novo DNA cytosine methyltransferase activities in mouse embryonic stem cells. Development.

[46] Amente S., Lania L., Majello B. (2013). The histone LSD1 demethylase in stemness and cancer transcription programs. Biochim. Et Biophys. Acta.

[47] Shi Y., Lan F., Matson C., Mulligan P., Whetstine J.R., Cole P.A., Casero R.A., Shi Y. (2004). Histone demethylation mediated by the nuclear amine oxidase homolog LSD1. Cell.

[48] Metzger E., Wissmann M., Yin N., Muller J.M., Schneider R., Peters A.H., Gunther T., Buettner R., Schule R. (2005). LSD1 demethylates repressive histone marks to promote androgen-receptor-dependent transcription. Nature.

[49] Cui S., Lim K.C., Shi L., Lee M., Jearawiriyapaisarn N., Myers G., Campbell A., Harro D., Iwase S., Trievel R.C. (2015). The LSD1 inhibitor RN-1 induces fetal hemoglobin synthesis and reduces disease pathology in sickle cell mice. Blood.

[50] Shi L., Cui S., Engel J.D., Tanabe O. (2013). Lysine-specific demethylase 1 is a therapeutic target for fetal hemoglobin induction. Nat. Med..

[51] Banzon V., Ibanez V., Vaitkus K., Ruiz M.A., Peterson K., DeSimone J., Lavelle D. (2011). siDNMT1 increases γ-globin expression in chemical inducer of dimerization (CID)-dependent mouse βYAC bone marrow cells and in baboon erythroid progenitor cell cultures. Exp. Hematol..

[52] Le C.Q., Myers G., Habara A., Jearawiriyapaisarn N., Murphy G.J., Chui D.H.K., Steinberg M.H., Engel J.D., Cui S. (2019). Inhibition of LSD1 by small molecule inhibitors stimulates fetal hemoglobin synthesis. Blood.

[53] Rivers A., Vaitkus K., Jagadeeswaran R., Ruiz M.A., Ibanez V., Ciceri F., Cavalcanti F., Molokie R.E., Saunthararajah Y., Engel J.D. (2018). Oral administration of the LSD1 inhibitor ORY-3001 increases fetal hemoglobin in sickle cell mice and baboons. Exp. Hematol..

[54] Gilmartin A.G., Groy A., Gore E.R., Atkins C., Long E.R., Montoute M.N., Wu Z., Halsey W., McNulty D.E., Ennulat D. (2021). In vitro and in vivo induction of fetal hemoglobin with a reversible and selective DNMT1 inhibitor. Haematologica.

[55] Amaya M., Desai M., Gnanapragasam M.N., Wang S.Z., Zu Zhu S., Williams D.C., Ginder G.D. (2013). Mi2β-mediated silencing of the fetal γ-globin gene in adult erythroid cells. Blood.

[56] Cammas F., Mark M., Dollé P., Dierich A., Chambon P., Losson R. (2000). Mice lacking the transcriptional corepressor TIF1beta are defective in early postimplantation development. Development.

[57] Miyagi S., Koide S., Saraya A., Wendt G.R., Oshima M., Konuma T., Yamazaki S., Mochizuki-Kashio M., Nakajima-Takagi Y., Wang C. (2014). The TIF1β-HP1 system maintains transcriptional integrity of hematopoietic stem cells. Stem Cell Rep..

[58] Hosoya T., Clifford M., Losson R., Tanabe O., Engel J.D. (2013). TRIM28 is essential for erythroblast differentiation in the mouse. Blood.

[59] Yu L., Jearawiriyapaisarn N., Lee M.P., Hosoya T., Wu Q., Myers G., Lim K.C., Kurita R., Nakamura Y., Vojtek A.B. (2018). BAP1 regulation of the key adaptor protein NCoR1 is critical for γ-globin gene repression. Genes Dev..

[60] Denslow S.A., Wade P.A. (2007). The human Mi-2/NuRD complex and gene regulation. Oncogene.

[61] Lee M.G., Wynder C., Bochar D.A., Hakimi M.-A., Cooch N., Shiekhattar R. (2006). Functional interplay between histone demethylase and deacetylase enzymes. Mol. Cell. Biol..

[62] Shi Y.-J., Matson C., Lan F., Iwase S., Baba T., Shi Y. (2005). Regulation of LSD1 histone demethylase activity by its associated factors. Mol. Cell.

[63] You A., Tong J.K., Grozinger C.M., Schreiber S.L. (2001). CoREST is an integral component of the CoREST- human histone deacetylase complex. Proc. Natl. Acad. Sci. USA.

[64] Cai Y., Geutjes E., de Lint K., Roepman P., Bruurs L., Yu L., Wang W., van Blijswijk J., Mohammad H., de Rink I. (2013). The NuRD complex cooperates with DNMTs to maintain silencing of key colorectal tumor suppressor genes. Oncogene.

[65] Lai A.Y., Wade P.A. (2011). Cancer biology and NuRD: A multifaceted chromatin remodelling complex. Nat. Rev. Cancer.

[66] Yu X., Azzo A., Bilinovich S.M., Li X., Dozmorov M., Kurita R., Nakamura Y., Williams D.C., Ginder G.D. (2019). Disruption of the MBD2-NuRD complex but not MBD3-NuRD induces high level HbF expression in human adult erythroid cells. Haematologica.

[67] Shang S., Li X., Azzo A., Truong T., Dozmorov M., Lyons C., Manna A.K., Williams D.C., Ginder G.D. (2023). MBD2a-NuRD binds to the methylated γ-globin gene promoter and uniquely forms a complex required for silencing of HbF expression. Proc. Natl. Acad. Sci. USA.

[68] Liang Y., Zhang X., Liu Y., Wang L., Ye Y., Tan X., Pu J., Zhang Q., Bao X., Wei X. (2021). GATA zinc finger domain-containing protein 2A (GATAD2A) deficiency reactivates fetal haemoglobin in patients with β-thalassaemia through impaired formation of methyl-binding domain protein 2 (MBD2)-containing nucleosome remodelling and deacetylation (NuRD) complex. Br. J. Haematol..

[69] Xu J., Bauer D.E., Kerenyi M.A., Vo T.D., Hou S., Hsu Y.J., Yao H., Trowbridge J.J., Mandel G., Orkin S.H. (2013). Corepressor-dependent silencing of fetal hemoglobin expression by BCL11A. Proc. Natl. Acad. Sci. USA.

[70] Martyn G.E., Wienert B., Yang L., Shah M., Norton L.J., Burdach J., Kurita R., Nakamura Y., Pearson R.C.M., Funnell A.P.W. (2018). Natural regulatory mutations elevate the fetal globin gene via disruption of BCL11A or ZBTB7A binding. Nat. Genet..

[71] Masuda T., Wang X., Maeda M., Canver M.C., Sher F., Funnell A.P., Fisher C., Suciu M., Martyn G.E., Norton L.J. (2016). Transcription factors LRF and BCL11A independently repress expression of fetal hemoglobin. Science.

[72] Sankaran V.G., Menne T.F., Xu J., Akie T.E., Lettre G., Van Handel B., Mikkola H.K., Hirschhorn J.N., Cantor A.B., Orkin S.H. (2008). Human fetal hemoglobin expression is regulated by the developmental stage-specific repressor BCL11A. Science.

[73] Cui S., Tanabe O., Lim K.-C., Xu H.E., Zhou X.E., Lin J.D., Shi L., Schmidt L., Campbell A., Shimizu R. (2014). PGC-1 coactivator activity is required for murine erythropoiesis. Mol. Cell. Biol..

[74] Sun Y., Habara A., Le C.Q., Nguyen N., Chen R., Murphy G.J., Chui D.H.K., Steinberg M.H., Cui S. (2022). Pharmacologic induction of PGC-1alpha stimulates fetal haemoglobin gene expression. Br. J. Haematol..

[75] Moriguchi T., Yamamoto M. (2014). A regulatory network governing Gata1 and Gata2 gene transcription orchestrates erythroid lineage differentiation. Int. J. Hematol..

[76] Gregory T., Yu C., Ma A., Orkin S.H., Blobel G.A., Weiss M.J. (1999). GATA-1 and erythropoietin cooperate to promote erythroid cell survival by regulating bcl-xL expression. Blood.

[77] Weiss M.J., Keller G., Orkin S.H. (1994). Novel insights into erythroid development revealed through in vitro differentiation of GATA-1 embryonic stem cells. Genes Dev..

[78] Weiss M.J., Orkin S.H. (1995). Transcription factor GATA-1 permits survival and maturation of erythroid precursors by preventing apoptosis. Proc. Natl. Acad. Sci. USA.

[79] Fujiwara Y., Browne C.P., Cunniff K., Goff S.C., Orkin S.H. (1996). Arrested development of embryonic red cell precursors in mouse embryos lacking transcription factor GATA-1. Proc. Natl. Acad. Sci. USA.

[80] Tanabe O., Shen Y., Liu Q., Campbell A.D., Kuroha T., Yamamoto M., Engel J.D. (2007). The TR2 and TR4 orphan nuclear receptors repress Gata1 transcription. Genes Dev..

[81] Crossley M., Tsang A.P., Bieker J.J., Orkin S.H. (1994). Regulation of the erythroid Kruppel-like factor (EKLF) gene promoter by the erythroid transcription factor GATA-1. J. Biol. Chem..

[82] Zhou D., Liu K., Sun C.-W., Pawlik K.M., Townes T.M. (2010). KLF1 regulates BCL11A expression and gamma- to beta-globin gene switching. Nat. Genet..

[83] Funnell A.P.W., Mak K.S., Twine N.A., Pelka G.J., Norton L.J., Radziewic T., Power M., Wilkins M.R., Bell-Anderson K.S., Fraser S.T. (2013). Generation of mice deficient in both KLF3/BKLF and KLF8 reveals a genetic interaction and a role for these factors in embryonic globin gene silencing. Mol. Cell. Biol..

[84] Borg J., Papadopoulos P., Georgitsi M., Gutiérrez L., Grech G., Fanis P., Phylactides M., Verkerk A.J.M.H., van der Spek P.J., Scerri C.A. (2010). Haploinsufficiency for the erythroid transcription factor KLF1 causes hereditary persistence of fetal hemoglobin. Nat. Genet..

